# Ergonomics and Biogeometry of Perforator/Propeller Flaps in the Lower Limb Reconstruction

**Published:** 2017-05

**Authors:** Mir Yasir, Adil Hafeez Wani, Haroon Rashid Zargar

**Affiliations:** Department of Plastic and Reconstructive Surgery, Skims, Srinagar, India

**Keywords:** Biogeometry, Ergonomics, Propeller flap, Perforator flap, Limb, Reconstraction

**DEAR EDITOR**

Reconstruction of lower limb defects is quite challenging in terms of scarcity of locally available flaps, poor wound healing and need for prolonged immobilization. Normally also there is decreased blood supply in the anterolateral aspect of leg and foot. Leg and foot is like a peninsula with no distal tissues available for proximal reconstruction. Also there is a paucity of loose tissue in leg and foot hence to reconstruct with a flap with least donor morbidity requires more expertise from the reconstructive surgeon. Local fasciocutaneous flaps, various muscle/ musculocutaneous flaps and free flaps were widely used. The era of perforator flaps began in 1989 describing their application and the great potential in harvesting of perforator flaps for lower limb defects.^1^^,^^2^

Perforator flap is defined as cutaneous paddle harvested on a direct cutaneous or septofasciocutaneous/musculocutaneous perforator which are rendered direct by periperforator dissection. Propeller flaps have two unequal blades (skin paddles) centered on the perforator and rotated on the single best perforator to the primary defect and the secondary blade potentially filling the secondary defect reducing tension on the perforator pedicle.^3^^-^^5^

 Perforator/propeller flaps enjoys a homogenized high vascularity. This is due to wide undermining and staging of the flap during the harvest itself leading to sympathectomy and contributing to increased blood supply. It has got the benefits of the musculocutaneous flaps minus the muscle. All steal phenonmenon due to undesired components like muscle, fascia, and fat is eliminated. This also contributes to the increased blood supply. Propeller flaps decrease the morbidity of the donor site by providing the small blade of tissue for partial reconstruction of the secondary defect. Also the standing cones and the reclining cones of the wide closing angles and unequal side respectively are not encountered in propeller flap which increases the aesthesis of the local reconstruction. It has all the benefits of local tissue with good color, thickness and texture match.^6^^,^^7^ This study assessed the ergonomics and biogeometry of perforator/propeller flaps in the lower limb reconstruction.

This prospective study was conducted between January 2009 and December 2013 in the Department Of Plastic Surgery of our institution. A total of 113 patients were included in this study. Only chronic posttraumatic and post excisional defects were included. Perforators flaps based on lateral calcaneal artery, superficial peroneal nerve artery, arcuate artery, anterior tibial artery, posterior tibial artery, ponten perforator, anterior recurrent introsseous artery, descending branch of ramus perforans, descending geniculate artery, superior gluteal artery, inferior gluteal artery, peroneal artery, lateral circumflex femoral artery, first dorsal metatarsal artery were the inclusion criteria. In each segment of lower limb minimum of three perforators were included in the study. Patients with diabetes, collagen vascular diseases, smoking or tobacco use in any form, vasculitis, immunocompromised, ulcers due to vascular insufficiency and unfavorable locoregional and general conditions were excluded from the study. 

Preoperatively patients were assessed clinically regarding evaluation for vascular insufficiency in delayed primary and secondary post traumatic defects. Following are the criteria for patient selection for flap cover. (i) There was minimal or no edema with healthy flat granulation, (ii) Good epithelizing front from the margin of the wound as shown by WBR edges (white-blue-red) (white-epibolic healing of epithelium with maceration blue ring- thin epithelium with underlying vasculature. red ring-centripetal granulation), (iii) Qualitative analysis of wound swab- no beta hemolytic *Streptococccus spp*, *Pseudomonas spp* grown, and (iv) With general and loco regional factors favoring the flap cover.

Those patients who fit into the above criteria , using hand held 10 mHz pencil Doppler, with 45 degree angulation towards onward flow perforators were located adjacent to the defect (Within 1-2 cm of the defect). If multiple perforators were located one with strong biphasic signal was chosen. Also local scars, vascularity, availability of loose tissues are taken into consideration. In situation where the perforator overlaid the underlying source vessel, when it was technically impossible to pick up the perforator by Doppler, we explored the perforator by single non-delineating incision.

We chose the single best perforator by the following criteria. (i) With visible pulsation, (ii) Size of facial defect through which perforator travels (wider the fascial defect better the perforator), (iii) Size of the perforator at fascial level after lignocaine spray and waiting for 10 min & finally, (iv) Those with one or two venae comitantes, and (v) Trial clamping (microvascular clamping) of the perforator and assessing perfusion of flap by superficial stab by using a eleven blade at the fartherest area of the delineated flap with dimensional allowance for choosing between perforator. This helps in identification of single best perforator.

In our experience, only in the gluteal region and especially in females the situation of the perforators differred by average area of 1.5 cm (This was related to the different posture during preoperative assessment on OT table and flabby skin). The direction of perforators which were running in the subcutaneous plane were relatively long distance downward and laterally directed especially in the gluteal region, also accounts for this variation. In rest of all areas, in the lower limb perforators were located exactly at the marked site. Considering this variation and to locate all visible perforators, we always began the surgery with single non delineating exploratory incision. After identification of all the visible perforators by above criteria, we chose the single best perforator and then biogeometry of the propeller flap was completed.

The farthest distance of the defect from the location of the single best perforator was measured to which 1.5 cm was added as an allowance for the primary contraction of the flap and 0.5 cm was added to the lesser dimension of the defect (breadth of the flap) for the similar purpose. From the single best perforator based on availability of the loose tissue in any direction the greatest dimension is projected and complete delineation of flap marked, steps of construction of flap was done as mentioned before. The large blade of flap was away from the defect whereas the small blade was closer to the defect. After the final inset, the smaller blade of the flap came to lie on the pedicle and part of the secondary defect adjacent to the primary defect. 

This step relieved the pressure over the pedicle and also generated closure of the secondary defect where it is feasible. Sometimes the flap was designed as trilobed flap when there was a circular defect. Wherein, the third lobe of the flap would come to lie in the part of the secondary defect, whereas, the smaller blade of flap (another lobe) would come to lie in the secondary defect of the third lobe. This biogeometric arrangement reduced the tension over closure and spread it over the secondary defect with secondary movement of interlobar skin. We always used 4x loupe magnification during elevation and preparation of the pedicle (periperforator) dissection. Peri perforator dissection was done around the cytoskeleton which carried the perforator bundle (artery, vein, lymphatics and possibly nerve twig). After thinning if required, removing the yellowish layer globular supra facial pad of fat and maintaining the granular whitish subdermal fat with aim of protecting undulating direct and indirect linking vessels of the perferosomes.^8^

We used hemostatic clips for supra fascial and subfascial and intramuscular branches of SBP. After satisfactory mobilization of the perforator, the flap was propelled towards the defect or incorporating primary interpolation or V-Y advancement, where V-Y advancement was used as primary movement the same was done with all steps of biogeometry of V-Y advancement. The propeller flap was done, critical assessment of clockwise/anticlockwise rotation which was causing the venous congestion and whether any kinking of perforator occurred and final inset of the flap was given too.

We found out by our experience that the venous congestion did not occur in one direction either clock or anticlock wise direction. We spent 5 minutes for assessment of venous congestion in each direction with trial inset. After determining satisfactory direction and no kinking/tension on perforator the final inset of flap was given. The following classification of cutaneous perforators was in our department: (i) Direct cutaneous perforators, (ii) Indirect cutaneous perforators, (iii) Neurocutaneous, (iv) Musculocutaneous, (v) Osteocutaneous, (vi) Glandulocutaneous and (vii) Septofascio cutaneous perforators (Table 1). The defective site and perforator from the preserved source vessel regarding length, width and surface area of the flap, limb length and ratio of flap length and width to the limb were presented in Table 2. 

**Table 1:** The classification of cutaneous perforators being followed in our department

**Perforator**	**Total no of cases**	**No of perforators (max)**	**No of perforators (min)**	**Size of perforators (mm)**	**Average size of flap supported on a single perforator (cm** ^2^ **)**
Septocutaneous perforators of lateral calcaneal artery	9	6	3	1.2	61.2
Neurocutaneous perforator from superficial peroneal nerve artery	6	8	4	1.5	130.65
Fasciocutaneous perforators from arcuate artery	6	5	1	1.7	52.96
Septofasciocutaneous perforators from Anterior tibial artery	13	7	3	2.2	161.32
Septofasciocutaneous perforators from Posterior tibial arter	8	6	4	1.7	184.0
Ponten perforator	4	2	1	2.1	201.8
Fasciocutaneous perforators from anterior interosseous recurrent artery	8	3	1	1.7	160.35
Perforators from Descending branch of ramus perforans	10	4	3	1.8	56.10
Septofasciocutaneous perforator from descending geniculate vessels	4	2	1	1.4	367.09
Propeller flap on Superior gluteal artery perforator	16	4	1	1.35	120.53
Propeller flap from Inferior gluteal artery perforator	10	4	2	1.8	97.44
Propeller flap on saphenous artery perforator	3	2	2	1.2	310.46
Propeller flap based on the peroneal artery proper perforator	14	7	0	1.8	148.71
Propeller flap based on the perforator from transverse branch of Lateral circumflex femoral artery	2	2	2	1.3	304.41

**Table 2:** The defective site and perforator from the preserved source vessel regarding length, width and surface area of the flap, limb length and ratio of flap length and width to the limb

**Defect site**	**Perforator from the preserved source vessel**	**Length of the flap (cm)**	**Limb length (cm)**	**Ratio of flap length to limb length**	**Width of flap (cm)**	**Circumference of limb (cm)**	**Ratio of flap width to limb circumference**	**Surface area of the flap (cm** ^2^ **)**
Foot	Lateral calcaneal artery	8.5	24.0	35.41	7.20	21.80	33.02	61.2
Leg	Superficial peroneal nerve artery	12.66	36.0	35.16	10.32	27.0	38.22	130.65
Foot	Arcuate artery	7.8	21.56	36.17	6.79	19.72	34.43	52.96
Leg	Anterior tibial artery	14.8	37.0	40.0	10.9	27.0	40.37	161.32
Leg	Posterior tibial artery	16	38.0	42.10	11.5	29.0	39.65	184.0
Leg	Ponten perforator	17.74	37.0	47.94	11.37	28.0	40.60	201.8
Leg	Anterior interosseous recurrent artery	13.80	36.0	38.33	11.62	28.0	41.50	160.35
Foot	Descending branch of ramus perforans	8.50	23.52	36.13	6.60	19.80	33.33	56.10
Thigh	Descending geniculate artery	19.61	48.23	40.68	18.72	45.96	40.73	367.09
Gluteal	Inferior gluteal artery	11.20	24.0	46.66	8.70	26.0	33.46	97.44
Gluteal	Superior gluteal artery	12.53	25.0	50.12	9.62	28.0	34.35	120.53
Thigh	Saphenous artery	17.56	42.72	41.10	17.68	44.89	39.38	310.46
Leg	Peroneal artery	15.72	36.0	43.66	9.46	26.0	36.38	148.71
Thigh	Transverse branch of lateral circumflex femoral artery	18.12	45.12	40.15	16.8	44.69	37.59	304.41

Hyperperfusion and increased blood flow exists in the perforator flaps as whole pressure head of source vessel was directed onto single best perforator and there was reduction of steal phenonmenon by other tissues like muscle and fascia and fat (where flap thinning is done). Flap combined the benefit of increased blood flow of musculocutaneous system minus the muscle. Single best perforator was chosen by size, visible and palpable pulsations and other parameters. This contributed to increased flow and larger flap harvest and 98% success rate by ready recruitment of more perferosomes opening the linkage vessels. Morbidity at donor site was almost nil. Even the source vessel with prominent cutaneous nerve, muscle and fascia were maintained at donor site.^3^^,^^9^^,^^10^

Systemic morbidity was also minimal as compared to bulky fasciocutaneous flaps, blood loss was also minimal and it is a microsurgical technique minus the microvascular anastamoses. Idiosyncracies of perforators in the lower limb were reported in gluteal region. We found out in all cases, the preoperative location of the perforators by 10 mhz pencil hand held Doppler did not correlate exactly with intraoperative finding.^9^ The location of perforators intraoperatively was 1.5-2.5 cm and an average of 1.8 cm from the preoperatively located site, explainable by the direction of vessels, directed downwards and laterally and the flabby buttocks with excess subcutaneous fat which were all responsible for this variation. At the donor site, no dog ears and contour deformities occurred most of the time when the secondary defects were closed primarily.^3^^,^^9^^,^^10^

Perforator from anterior tibial vessels around the ankle region and posterior tibial perforator around the ankle region because of superficial nature of source vessel perforator could not be picked up by preoperative examination by Doppler. In this region with respect to defect, we first put exploratory incision identify the perforator and finally designed flap dimension based on single best perforator. By definition these can be called as Adhoc perforator /propeller flap.^3^^,^^9^^,^^10^

Considering the high homogenized blood supply in all perforator/propeller flaps because of symphatectomy with staging and delay occurred on raising of the flap. With added advantage of thinning to the desired extend reduce the steal phenomenon by the other tissues. This paves the way for robust flap with respect to the blood supply with tolerance for even 180 degree rotation about the pedicle. A small blade of the propeller flap sets into the secondary defect which gives tension less closure. Periperforator dissection to the desired extent is performed from facial exit of perforator to intermuscular plane under loupe magnification is technically though demanding is easily performed with short and steep learning curve. 

The periperforator dissection is performed by clipping the supra/subfacial, intramuscular perforators using hemostatic clips. The biogeometrical steps and planning in reverse are performed as mentioned previously. From the analysis of data we have come into reasonable conclusion based on regional single best perforator up to one third of length of the segment of lower limb can be safe length of the flap and up to one third of circumference of the segment of lower limb can be the safe breadth of the perforator/propeller flap. In the gluteal region up to one third of greatest dimension of the buttocks can be safe greatest dimension of the flap. 

By this study we are trying to answer the baffling question as to what is the maximum safe size of the flap that can be harvested on single best perforator. In the lower limb area with the regression analysis of available data we have come into the reasonable conclusion that up to one third of length of the segment of lower limb can be the safe length of the flap and up to one third of circumference of the segment of lower limb can be the safe breadth of the perforator/propeller flap. In the gluteal region up to one third of greatest dimension of the buttocks can be safe greatest dimension of the flap. All the flaps settled well with 2% necrosis rate.

## CONFLICT OF INTEREST

The authors declare no conflict of interest.
